# Poly[di-μ_2_-chlorido-dichlorido(μ_3_-di­methyl sulfoxide-κ^3^ *O*:*O*:*S*)(μ_2_-di­methyl sulfoxide-κ^2^ *O*:*S*)ruthenium(III)sodium]

**DOI:** 10.1107/S1600536810007063

**Published:** 2010-03-03

**Authors:** Zdeněk Trávníček, Miroslava Matiková-Maľarová

**Affiliations:** a Department of Inorganic Chemistry, Faculty of Science, Palacký University, Třída 17. listopadu 12, CZ-771 46 Olomouc, Czech Republic

## Abstract

The structure of the title compound, [NaRuCl_4_(C_2_H_6_OS)_2_]_*n*_, comprises centrosymmetric [RuCl_2_(DMSO)Na(DMSO)Cl_2_Ru] units (DMSO is dimethyl sulfoxide, C_2_H_6_OS), with two Ru atoms, each lying on a crystallographic centre of inversion, connected *via* Na atoms, DMSO and chloride ligands into a two-dimensional (110) array. Both Ru^III^ atoms are octa­hedrally coordinated by four chloride ligands in the equatorial plane and by two DMSO mol­ecules in apical positions within a RuCl_4_S_2_ donor set. The Na atom is surrounded by three chloride anions and three O atoms derived from three DMSO mol­ecules, with the resulting Cl_3_O_3_ donor set defining an octa­hedron. The crystal structure is further stabilized by inter­atomic inter­actions of the types C⋯Cl [C—Cl = 3.284 (2) Å], C—H⋯Cl [C⋯Cl = 3.903 (3) Å] and C—H⋯O [C⋯O = 3.376 (3) Å].

## Related literature

For structures of similar ruthenium complexes, see: Alessio *et al.* (1993 ▶); Piggot *et al.* (2004 ▶); Anderson *et al.* (2007 ▶). For Na—O and Na—Cl distances in related structures, see: Alessio *et al.* (1991 ▶); Iengo *et al.* (1999 ▶).
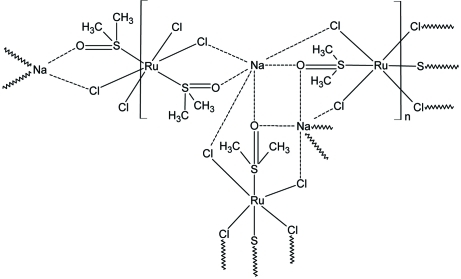


## Experimental

### 

#### Crystal data

[NaRuCl_4_(C_2_H_6_OS)_2_]
*M* *_r_* = 422.12
Monoclinic, 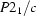

*a* = 11.9042 (3) Å
*b* = 8.0692 (2) Å
*c* = 13.7873 (3) Å
β = 98.470 (2)°
*V* = 1309.93 (5) Å^3^
*Z* = 4
Mo *K*α radiation
μ = 2.34 mm^−1^
*T* = 120 K
0.20 × 0.20 × 0.15 mm

#### Data collection

Oxford Diffraction Xcalibur2 CCD diffractometer
Absorption correction: multi-scan (*CrysAlis RED*; Oxford Diffraction, 2007 ▶) *T*_min_ = 0.652, *T*_max_ = 0.721
10197 measured reflections
2300 independent reflections
2105 reflections with *I* > 2σ(*I*)
*R*_int_ = 0.021

#### Refinement

*R*[*F* ^2^ > 2σ(*F* ^2^)] = 0.018
*wR*(*F* ^2^) = 0.050
*S* = 1.15
2300 reflections
134 parameters
H-atom parameters constrained
Δρ_max_ = 0.71 e Å^−3^
Δρ_min_ = −0.36 e Å^−3^

### 

Data collection: *CrysAlis CCD* (Oxford Diffraction, 2007 ▶); cell refinement: *CrysAlis RED* (Oxford Diffraction, 2007 ▶); data reduction: *CrysAlis RED*; program(s) used to solve structure: *SHELXS97* (Sheldrick, 2008 ▶); program(s) used to refine structure: *SHELXL97* (Sheldrick, 2008 ▶); molecular graphics: *DIAMOND* (Brandenburg, 2006 ▶); software used to prepare material for publication: *SHELXL97*.

## Supplementary Material

Crystal structure: contains datablocks I, global. DOI: 10.1107/S1600536810007063/tk2632sup1.cif

Structure factors: contains datablocks I. DOI: 10.1107/S1600536810007063/tk2632Isup2.hkl

Additional supplementary materials:  crystallographic information; 3D view; checkCIF report

## Figures and Tables

**Table 1:** Selected bond lengths (Å)

Ru1—S1	2.3350 (5)
Ru1—Cl1	2.3509 (5)
Ru1—Cl2	2.3551 (5)
Na1—O2	2.2974 (18)
Na1—O1	2.4105 (17)
Na1—O1^i^	2.4155 (18)
Na1—Cl4^ii^	2.7773 (11)
Na1—Cl1^iii^	2.8769 (10)
Na1—Cl2^iv^	2.9374 (10)
Ru2—Cl3	2.3353 (6)
Ru2—S2	2.3373 (6)
Ru2—Cl4	2.3663 (6)

Symmetry codes: (i) 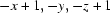; (ii) 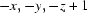; (iii) 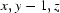; (iv) 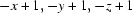.

**Fig. 1.**
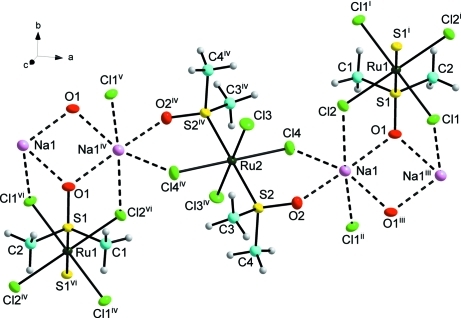
Part of the crystal structure of the title compound (I). The non-H atoms are drawn as 70% probability displacement ellipsoids. Symmetry codes: (i) -x+1, -y+1, -z+1; (ii) x, y+1, z; (iii) -x+1, -y, -z+1; (iv) -x, -y, 1-z; (v) x, y-1, z; (vi) -1+x, -1+y, z.

**Fig. 2.**
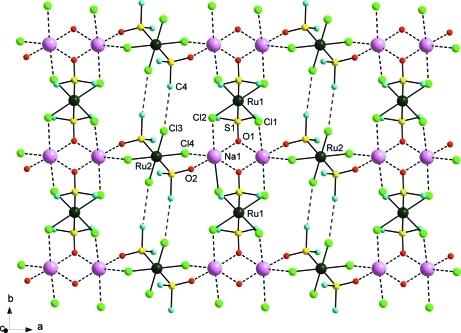
Part of the crystal structure of (I), showing the formation of the two dimensional array (a view along the c axis). Dashed lines represent non-covalent interactions of the C···Cl, Na···O and Na···Cl types. The H-atoms have been omitted for clarity. Symmetry code: (ii) x, 1+y, z.

**Fig. 3.**
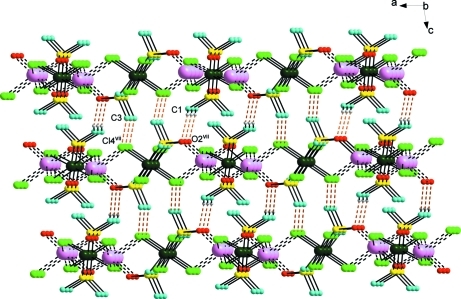
Part of the crystal structure of (I), showing the formation of the two dimensional array (a view along the b axis). The H-atoms have been omitted for clarity. Dashed lines represent interactions of the C···Cl and C—H···O types. Symmetry code: (vii) x, 1/2-y, -1/2+z.

## supplementary crystallographic information

### Comment

The title complex, (I), was prepared as a part of our study of simple ruthenium(III) complexes of the composition M[RuCl_4_(DMSO)_2_] with the different types of inorganic and organic cations [M]. In the crystal structure of (I), the centrosymmetric [RuCl_2_(DMSO)Na(DMSO)Cl_2_Ru] units are present; each ruthenium atom lies on a crystallographic centre of inversion whereas all other atoms lie in general positions. Each of the Ru^III^ atoms is hexacoordinated by two sulphur atoms from two DMSO molecules in apical positions and four chlorido ligands in an equatorial plane (Fig. 1). Each ruthenium atom exists within an octahedral *trans*-RuCl_4_S_2_ donor set.

Both octahedra exhibit similar Ru—S bond lengths [Ru1—S1 = 2.3350 (5) and Ru2—S2= 2.3373 (6) Å] in contrast to the Ru—Cl bond lengths, which exhibit similar values in the Ru1 octahedron [2.3551 (5) and 2.3509 (5) Å] but differ significantly in the Ru2 octahedron [2.3353 (6) and 2.3663 (6) Å]. The Ru—Cl bond lengths within the [RuCl_2_(DMSO)Na(DMSO)Cl_2_Ru] unit do not differ markedly from those observed in Na[*trans*-RuCl_4_(DMSO)(pyr)].DMSO, where pyr = 1,4-pyrazine, [Ru—Cl = (2.3395 (8) - 2.3754 (7) Å] (Anderson *et al.*, 2007) and in Na_2_[{*trans*-RuCl_4_(DMSO)}_2_(µ-pyrimidine)], where the Ru—Cl bond lengths are in the range of 2.338 (2) - 2.361 (2) Å (Iengo *et al.*, 1999), while the Ru—S distances in (I) are slightly longer than those observed in the above-mentioned complexes (Ru—S are in the range of 2.281 (2) - 2.3027 (7) Å).

The Na cation has six atoms in its closest octahedrally coordinated environment with the resulting Cl_3_O_3_ donor set and it is surrounded by six donor interactions, represented by dashed lines (Figs. 1 and 2). The distance between the two nearest Na atoms equals 3.4968 (19) Å. The distances of the Na—O and Na—Cl donor interactions vary from 2.2974 (18) to 2.4155 (18) Å, and from 2.7773 (11) to 2.9374 (10) Å, respectively. Similar values of the Na—O distances [2.272 (4) - 2.418 (4) Å] were found in Na[*trans*-RuCl_4_(DMSO)(NH_3_)].DMSO (Alessio *et al.*, 1993) and similar Na—Cl distances [2.776 (2) - 2.922 (2) Å] were observed in Na[*trans*-RuCl_4_(DMSO)(pyr)].DMSO (Anderson *et al.*, 2007).

The *trans*-[Ru(DMSO)_2_Cl_4_] complex anion is connected to four sodium cations, two DMSO molecules and four chlorido ligands into infinite two dimensional array in the *ab* plane (Fig. 2). The crystal strucure of (I) is furter stabilized by non-bonding interactions of the C···Cl type [C4···Cl3^ii^ is 3.284 (2) Å (ii = x, y+1, z)], which connect two Ru2 anions in the *b* direction (Fig. 2). The planes are cross connected, in the *c *direction, by the weak C···Cl [C3—Cl4^vii^ is 3.903 (3) Å] and C—H···O [C1···O2^vii^ is 3.376 (3) Å (vii = x, 1/2-y, -1/2+z)] type interactions (Fig. 3).

### Experimental

The title complex was prepared by a slightly modified literature procedure (Alessio *et al.*, 1991). The salt [H^+^(DMSO)_2_][*trans*-Ru(DMSO)_2_Cl_4_] (1 mmol) was dissolved in a mixture of ethanol (20 ml) and distilled water (0.3 ml). The orange solution was filtered, and then, an aqueous solution (0.3 ml) of NaCl (0.07 g, 2.8 mmol) was added during stirring. The yellow precipitate which formed after several minutes was filtered off, washed with cold acetone, and dried in air (yield 50%). Single crystals were obtained from the filtrate by slow evaporation after several days.

### Refinement

All H-atoms were located in difference maps and refined using a riding model, with C–H distances of 0.98 Å, and with U_iso_(H) = 1.5U_eq_(C).

### Crystal data

**Table:**

[NaRuCl_4_(C_2_H_6_OS)_2_]	*F*(000) = 828
*M**_r_* = 422.12	*D*_x_ = 2.140 Mg m^−^^3^
Monoclinic, *P*2_1_/*c*	Mo *K*α radiation, λ = 0.71073 Å
Hall symbol: -P 2ybc	Cell parameters from 10079 reflections
*a* = 11.9042 (3) Å	θ = 2.9–32.0°
*b* = 8.0692 (2) Å	µ = 2.34 mm^−^^1^
*c* = 13.7873 (3) Å	*T* = 120 K
β = 98.470 (2)°	Prism, orange
*V* = 1309.93 (5) Å^3^	0.20 × 0.20 × 0.15 mm
*Z* = 4	

### Data collection

**Table:**

Oxford Diffraction Xcalibur2 CCD diffractometer	2300 independent reflections
Radiation source: fine-focus sealed tube	2105 reflections with *I* > 2σ(*I*)
Enhance (Mo) X-ray Source	*R*_int_ = 0.021
Detector resolution: 8.3611 pixels mm^-1^	θ_max_ = 25.0°, θ_min_ = 2.9°
rotation method, ω–scan	*h* = −14→12
Absorption correction: multi-scan (*CrysAlis RED*; Oxford Diffraction, 2007)	*k* = −9→9
*T*_min_ = 0.652, *T*_max_ = 0.721	*l* = −14→16
10197 measured reflections	

### Refinement

**Table:**

Refinement on *F*^2^	Primary atom site location: structure-invariant direct methods
Least-squares matrix: full	Secondary atom site location: difference Fourier map
*R*[*F*^2^ > 2σ(*F*^2^)] = 0.018	Hydrogen site location: inferred from neighbouring sites
*wR*(*F*^2^) = 0.050	H-atom parameters constrained
*S* = 1.15	*w* = 1/[σ^2^(*F*_o_^2^) + (0.0269*P*)^2^ + 0.8654*P*] where *P* = (*F*_o_^2^ + 2*F*_c_^2^)/3
2300 reflections	(Δ/σ)_max_ = 0.001
134 parameters	Δρ_max_ = 0.71 e Å^−^^3^
0 restraints	Δρ_min_ = −0.36 e Å^−^^3^

### Special details

**Table:**

Geometry. All esds (except the esd in the dihedral angle between two l.s. planes) are estimated using the full covariance matrix. The cell esds are taken into account individually in the estimation of esds in distances, angles and torsion angles; correlations between esds in cell parameters are only used when they are defined by crystal symmetry. An approximate (isotropic) treatment of cell esds is used for estimating esds involving l.s. planes.
Refinement. Refinement of *F*^2^ against ALL reflections. The weighted *R*-factor wR and goodness of fit *S* are based on *F*^2^, conventional *R*-factors *R* are based on *F*, with *F* set to zero for negative *F*^2^. The threshold expression of *F*^2^ > σ(*F*^2^) is used only for calculating *R*-factors(gt) etc. and is not relevant to the choice of reflections for refinement. *R*-factors based on *F*^2^ are statistically about twice as large as those based on *F*, and *R*- factors based on ALL data will be even larger.

### Fractional atomic coordinates and isotropic or equivalent isotropic displacement parameters (Å^2^)

**Table:**

	*x*	*y*	*z*	*U*_iso_*/*U*_eq_	
Ru1	0.5000	0.5000	0.5000	0.00777 (8)	
S1	0.45936 (5)	0.30580 (7)	0.37416 (4)	0.00948 (13)	
Cl1	0.35603 (5)	0.67940 (7)	0.42755 (4)	0.01284 (13)	
Na1	0.35677 (8)	0.00303 (10)	0.51520 (7)	0.0144 (2)	
O1	0.47177 (13)	0.12823 (19)	0.40510 (11)	0.0125 (3)	
C1	0.31978 (19)	0.3274 (3)	0.30958 (17)	0.0148 (5)	
H1A	0.3050	0.2387	0.2608	0.022*	
H1B	0.3125	0.4351	0.2765	0.022*	
H1C	0.2648	0.3202	0.3558	0.022*	
Ru2	0.0000	0.0000	0.5000	0.00862 (9)	
S2	0.12479 (5)	−0.14419 (7)	0.61717 (4)	0.01206 (13)	
Cl2	0.63676 (5)	0.64639 (7)	0.42686 (4)	0.01285 (13)	
O2	0.24699 (13)	−0.0998 (2)	0.62514 (11)	0.0147 (4)	
C2	0.5426 (2)	0.3346 (3)	0.27921 (16)	0.0145 (5)	
H2A	0.5163	0.2593	0.2249	0.022*	
H2B	0.6224	0.3112	0.3042	0.022*	
H2C	0.5352	0.4494	0.2559	0.022*	
Cl3	0.07120 (5)	0.23663 (7)	0.58529 (4)	0.01694 (14)	
C3	0.0887 (2)	−0.1226 (4)	0.73698 (18)	0.0224 (6)	
H3A	0.1455	−0.1794	0.7841	0.034*	
H3B	0.0138	−0.1718	0.7390	0.034*	
H3C	0.0868	−0.0048	0.7539	0.034*	
Cl4	−0.14497 (5)	−0.01787 (7)	0.59997 (4)	0.01570 (14)	
C4	0.1151 (2)	−0.3619 (3)	0.6015 (2)	0.0262 (6)	
H4A	0.1588	−0.4168	0.6583	0.039*	
H4B	0.1458	−0.3934	0.5420	0.039*	
H4C	0.0354	−0.3960	0.5956	0.039*	

### Atomic displacement parameters (Å^2^)

**Table:**

	*U*^11^	*U*^22^	*U*^33^	*U*^12^	*U*^13^	*U*^23^
Ru1	0.00907 (15)	0.00689 (15)	0.00724 (14)	0.00048 (9)	0.00081 (11)	0.00072 (9)
S1	0.0118 (3)	0.0075 (3)	0.0088 (3)	0.0004 (2)	0.0006 (2)	0.0000 (2)
Cl1	0.0137 (3)	0.0105 (3)	0.0133 (3)	0.0032 (2)	−0.0017 (2)	0.0002 (2)
Na1	0.0131 (5)	0.0128 (5)	0.0173 (5)	−0.0001 (3)	0.0023 (4)	0.0027 (4)
O1	0.0165 (9)	0.0073 (8)	0.0136 (8)	0.0005 (6)	0.0015 (7)	0.0005 (6)
C1	0.0134 (12)	0.0148 (12)	0.0148 (12)	0.0005 (10)	−0.0027 (10)	−0.0016 (10)
Ru2	0.00868 (15)	0.00670 (15)	0.01060 (15)	−0.00093 (9)	0.00177 (11)	−0.00048 (9)
S2	0.0113 (3)	0.0106 (3)	0.0142 (3)	−0.0004 (2)	0.0014 (2)	0.0025 (2)
Cl2	0.0140 (3)	0.0126 (3)	0.0126 (3)	−0.0021 (2)	0.0042 (2)	0.0007 (2)
O2	0.0108 (8)	0.0183 (9)	0.0148 (8)	0.0001 (7)	0.0013 (7)	0.0030 (7)
C2	0.0195 (13)	0.0135 (12)	0.0110 (11)	−0.0006 (10)	0.0042 (10)	−0.0009 (9)
Cl3	0.0202 (3)	0.0107 (3)	0.0194 (3)	−0.0040 (2)	0.0013 (3)	−0.0048 (2)
C3	0.0176 (13)	0.0349 (16)	0.0147 (12)	0.0010 (11)	0.0022 (11)	0.0066 (11)
Cl4	0.0116 (3)	0.0220 (3)	0.0142 (3)	−0.0003 (2)	0.0040 (2)	0.0019 (2)
C4	0.0267 (15)	0.0113 (13)	0.0383 (16)	0.0002 (11)	−0.0032 (13)	0.0039 (11)

### Geometric parameters (Å, °)

**Table:**

Ru1—S1	2.3350 (5)	Ru2—Cl3^iv^	2.3353 (6)
Ru1—S1^i^	2.3351 (5)	Ru2—Cl3	2.3353 (6)
Ru1—Cl1	2.3509 (5)	Ru2—S2^iv^	2.3373 (6)
Ru1—Cl1^i^	2.3509 (5)	Ru2—S2	2.3373 (6)
Ru1—Cl2^i^	2.3551 (5)	Ru2—Cl4^iv^	2.3663 (6)
Ru1—Cl2	2.3551 (5)	Ru2—Cl4	2.3663 (6)
S1—O1	1.4962 (16)	S2—O2	1.4863 (16)
S1—C2	1.770 (2)	S2—C4	1.772 (3)
S1—C1	1.774 (2)	S2—C3	1.776 (2)
Cl1—Na1^ii^	2.8769 (10)	Cl2—Na1^i^	2.9374 (10)
Na1—O2	2.2974 (18)	C2—H2A	0.9800
Na1—O1	2.4105 (17)	C2—H2B	0.9800
Na1—O1^iii^	2.4155 (18)	C2—H2C	0.9800
Na1—Cl4^iv^	2.7773 (11)	C3—H3A	0.9800
Na1—Cl1^v^	2.8769 (10)	C3—H3B	0.9800
Na1—Cl2^i^	2.9374 (10)	C3—H3C	0.9800
Na1—Na1^iii^	3.4968 (19)	Cl4—Na1^iv^	2.7772 (11)
O1—Na1^iii^	2.4156 (18)	C4—H4A	0.9800
C1—H1A	0.9800	C4—H4B	0.9800
C1—H1B	0.9800	C4—H4C	0.9800
C1—H1C	0.9800		
			
S1—Ru1—S1^i^	179.999 (1)	S1—C1—H1B	109.5
S1—Ru1—Cl1	92.250 (19)	H1A—C1—H1B	109.5
S1^i^—Ru1—Cl1	87.751 (19)	S1—C1—H1C	109.5
S1—Ru1—Cl1^i^	87.751 (19)	H1A—C1—H1C	109.5
S1^i^—Ru1—Cl1^i^	92.248 (19)	H1B—C1—H1C	109.5
Cl1—Ru1—Cl1^i^	180.0	Cl3^iv^—Ru2—Cl3	179.999 (1)
S1—Ru1—Cl2^i^	84.313 (19)	Cl3^iv^—Ru2—S2^iv^	84.97 (2)
S1^i^—Ru1—Cl2^i^	95.687 (19)	Cl3—Ru2—S2^iv^	95.03 (2)
Cl1—Ru1—Cl2^i^	89.091 (19)	Cl3^iv^—Ru2—S2	95.03 (2)
Cl1^i^—Ru1—Cl2^i^	90.909 (19)	Cl3—Ru2—S2	84.97 (2)
S1—Ru1—Cl2	95.686 (19)	S2^iv^—Ru2—S2	179.999 (1)
S1^i^—Ru1—Cl2	84.314 (19)	Cl3^iv^—Ru2—Cl4^iv^	89.90 (2)
Cl1—Ru1—Cl2	90.909 (19)	Cl3—Ru2—Cl4^iv^	90.10 (2)
Cl1^i^—Ru1—Cl2	89.091 (19)	S2^iv^—Ru2—Cl4^iv^	90.63 (2)
Cl2^i^—Ru1—Cl2	180.0	S2—Ru2—Cl4^iv^	89.37 (2)
O1—S1—C2	107.12 (10)	Cl3^iv^—Ru2—Cl4	90.10 (2)
O1—S1—C1	106.43 (10)	Cl3—Ru2—Cl4	89.90 (2)
C2—S1—C1	101.59 (11)	S2^iv^—Ru2—Cl4	89.37 (2)
O1—S1—Ru1	115.46 (7)	S2—Ru2—Cl4	90.63 (2)
C2—S1—Ru1	112.59 (8)	Cl4^iv^—Ru2—Cl4	180.0
C1—S1—Ru1	112.54 (8)	O2—S2—C4	107.04 (12)
Ru1—Cl1—Na1^ii^	115.06 (3)	O2—S2—C3	106.03 (11)
O2—Na1—O1	176.27 (7)	C4—S2—C3	100.97 (14)
O2—Na1—O1^iii^	93.79 (6)	O2—S2—Ru2	116.54 (7)
O1—Na1—O1^iii^	87.14 (6)	C4—S2—Ru2	112.72 (10)
O2—Na1—Cl4^iv^	80.56 (5)	C3—S2—Ru2	112.19 (9)
O1—Na1—Cl4^iv^	100.01 (5)	Ru1—Cl2—Na1^i^	111.02 (3)
O1^iii^—Na1—Cl4^iv^	156.08 (5)	S2—O2—Na1	133.14 (10)
O2—Na1—Cl1^v^	88.98 (5)	S1—C2—H2A	109.5
O1—Na1—Cl1^v^	94.75 (5)	S1—C2—H2B	109.5
O1^iii^—Na1—Cl1^v^	75.12 (4)	H2A—C2—H2B	109.5
Cl4^iv^—Na1—Cl1^v^	81.52 (3)	S1—C2—H2C	109.5
O2—Na1—Cl2^i^	99.39 (5)	H2A—C2—H2C	109.5
O1—Na1—Cl2^i^	76.89 (4)	H2B—C2—H2C	109.5
O1^iii^—Na1—Cl2^i^	108.18 (5)	S2—C3—H3A	109.5
Cl4^iv^—Na1—Cl2^i^	95.69 (3)	S2—C3—H3B	109.5
Cl1^v^—Na1—Cl2^i^	170.66 (4)	H3A—C3—H3B	109.5
O2—Na1—Na1^iii^	137.18 (6)	S2—C3—H3C	109.5
O1—Na1—Na1^iii^	43.63 (4)	H3A—C3—H3C	109.5
O1^iii^—Na1—Na1^iii^	43.51 (4)	H3B—C3—H3C	109.5
Cl4^iv^—Na1—Na1^iii^	138.71 (5)	Ru2—Cl4—Na1^iv^	110.05 (3)
Cl1^v^—Na1—Na1^iii^	83.09 (3)	S2—C4—H4A	109.5
Cl2^i^—Na1—Na1^iii^	93.39 (4)	S2—C4—H4B	109.5
S1—O1—Na1	122.65 (9)	H4A—C4—H4B	109.5
S1—O1—Na1^iii^	126.22 (9)	S2—C4—H4C	109.5
Na1—O1—Na1^iii^	92.86 (6)	H4A—C4—H4C	109.5
S1—C1—H1A	109.5	H4B—C4—H4C	109.5

Symmetry codes: (i) −*x*+1, −*y*+1, −*z*+1; (ii) *x*, *y*+1, *z*; (iii) −*x*+1, −*y*, −*z*+1; (iv) −*x*, −*y*, −*z*+1; (v) *x*, *y*−1, *z*.

## References

[bb1] Alessio, E., Balducci, G., Calligaris, M., Costa, G., Attia, G. M. & Mestroni, G. (1993). *Inorg. Chim. Acta*, **30**, 609–618.

[bb2] Alessio, E., Balducci, G., Lutman, A., Mestroni, G., Calligaris, M. & Attia, G. M. (1991). *Inorg. Chim. Acta*, **203**, 205–217.

[bb3] Anderson, C. M., Herman, A. & Rochon, F. D. (2007). *Polyhedron*, **26**, 3661–3668.

[bb4] Brandenburg, K. (2006). *DIAMOND* Crystal Impact GbR, Bonn, Germany.

[bb5] Iengo, E., Mestroni, G., Geremia, S., Calligaris, M. & Alessio, E. (1999). *J. Chem. Soc. Dalton Trans.* pp. 3361–3371.

[bb6] Oxford Diffraction (2007). *CrysAlis CCD* and *CrysAlis RED* Oxford Diffraction Ltd, Abingdon, England.

[bb7] Piggot, P. M. T., Hall, L. A., White, A. J. P. & Williams, D. J. (2004). *Inorg. Chim. Acta*, **357**, 250–258.

[bb8] Sheldrick, G. M. (2008). *Acta Cryst.* A**64**, 112–122. 10.1107/S0108767307043930 18156677

